# Using Elastographic Stiffness to Improve Risk Stratification in Medullary Thyroid Carcinoma

**DOI:** 10.3390/diagnostics15212742

**Published:** 2025-10-29

**Authors:** Monica Latia, Andreea Bena, Octavian Constantin Neagoe, Dana Stoian

**Affiliations:** 1 Department of Doctoral Studies, Victor Babes University of Medicine and Pharmacy, 300041 Timisoara, Romania; monica.latia@umft.ro; 2 Dr. D Medical Center, Center for Advanced Ultrasound Evaluation, 300029 Timisoara, Romaniastoian.dana@umft.ro (D.S.); 3 Center of Molecular Research in Nephrology and Vascular Disease, Faculty of Medicine, Victor Babes University of Medicine and Pharmacy, 300041 Timisoara, Romania; 4 2nd Department of Internal Medicine, Victor Babes University of Medicine and Pharmacy, 300041 Timisoara, Romania; 5 Endocrinology Unit, Pius Brinzeu Emergency Clinical Hospital, 300723 Timisoara, Romania; 6 1st Department of Surgery, Victor Babes University of Medicine and Pharmacy, 300041 Timisoara, Romania; 7 Second Clinic of General Surgery and Surgical Oncology, Emergency Clinical Municipal Hospital, 300254 Timisoara, Romania

**Keywords:** thyroid neoplasms, elastography, calcitonin, medullary carcinoma, lymph node metastasis

## Abstract

**Background/Objectives**: Medullary thyroid carcinoma (MTC) poses diagnostic challenges due to its sonographic similarity to benign nodules and the modest sensitivity of conventional ultrasound (US) and TIRADS-based risk stratification. Elastography, using strain (SE) and shear-wave (SWE) techniques, has demonstrated high accuracy in papillary thyroid carcinoma (PTC) but remains underexplored in MTC. This study investigates whether elastographic stiffness measurements can enhance diagnostic precision for MTC when combined with conventional US. **Methods**: We retrospectively analyzed 20 nodules confirmed as MTC by pathology after surgical resection (January 2013–June 2024) and evaluated via conventional US, SE, and SWE at a specialized endocrinology center. Elasticity scores (ES) and Emean/Emax values were compared with US features, TIRADS categories, calcitonin levels, nodule size, and lymph node status. **Results**: Qualitative SE showed a mean ES of 3.2 (55% ES 4, 20% ES 3), while 87.5% of nodules exceeded an SWE Emean cutoff of 30.5 kPa, indicating increased stiffness in most MTC nodules and outperforming TIRADS, where only 60% were high-risk. Moderate correlations were found between calcitonin and nodule size (r = 0.52, *p* = 0.018) and between ES and size (r = 0.48, *p* = 0.034), but calcitonin did not correlate with ES (r = 0.07, *p* = 0.768). Nodules with suspicious lymph nodes showed higher Emean and ES trends, though not significant. **Conclusions**: Elastography identifies increased stiffness in MTC, challenging its “soft” classification, and improves risk stratification beyond TIRADS. We suggest integrating elastography as a complementary tool alongside TIRADS to guide fine-needle aspiration, without replacing calcitonin or cytology. Prospective multicenter studies are needed to validate thresholds and optimize multimodal risk assessment in MTC.

## 1. Introduction

Thyroid nodules are very common in the general population, with detection rates increasing due to the widespread use of high-resolution imaging techniques. Up to 60% of adults may harbor one or more thyroid nodules, with higher prevalence observed in women and the elderly [1,2]. While the majority of these nodules are benign, the risk of malignancy (ROM) is 5–15%, depending on clinical context. The incidence of thyroid cancer has risen globally over the past few decades, due to enhanced detection methods and greater awareness [3]. Thyroid cancers are classified into several types, including papillary (PTC), follicular (FTC), medullary, and anaplastic carcinomas. Medullary thyroid carcinoma (MTC) is a rare subtype, accounting for approximately 1–5% of all thyroid cancers and 1–2% of all thyroid nodules [4,5].

MTC is a neuroendocrine tumor derived from parafollicular C-cells, which produce calcitonin. Early diagnosis is critical, as MTC often metastasizes early to lymph nodes and distant sites, significantly affecting prognosis [6]. While ultrasound (US) is the first-line imaging modality for thyroid nodules, current risk stratification systems (RSSs), such as ACR-TIRADS, ATA, EU-TIRADS, Kwak, and C-TIRADS, are less effective in identifying MTC compared to PTC. Studies show that only about 55–60% of MTCs are classified in a high-suspicion category or recommended for fine-needle aspiration (FNA), meaning a substantial proportion may be missed [7,8,9]. Moreover, fewer than 10% of MTCs exhibit four or more classic high-risk US features simultaneously. This discrepancy arises because MTC often presents with features such as wider-than-tall shape and increased vascularity, which are underrepresented in RSS scoring. Consequently, relying solely on RSSs may lead to delayed diagnosis [10,11].

Elastography enhances conventional US by providing valuable information on tissue stiffness, improving the differentiation of benign and malignant thyroid nodules by considering stiffness as an indicator of malignancy. Among the elastography techniques, strain elastography (SE) evaluates tissue deformation in response to external compression, offering qualitative and semi-quantitative data. Shear-wave elastography (SWE), on the other hand, generates and measures the velocity of shear waves within the tissue, providing objective and quantitative stiffness measurements [12,13]. Both methods complement the morphological assessment provided by conventional US, increasing the overall diagnostic accuracy. Recognizing their clinical utility, the World Federation for Ultrasound in Medicine and Biology (WFUMB) endorses the use of both SE and SWE in the evaluation of thyroid nodules [14].

However, elastographic evaluation of MTC presents different challenges, as these tumors often appear soft or heterogeneous on both SE and SWE, frequently leading to false-negative results. This is largely due to their high amyloid content and low fibrosis, which reduce the diagnostic reliability of SE, so that up to 60% of MTC nodules are misclassified as benign. While SWE provides quantitative data and typically reveals stiffness values higher than normal thyroid tissue but lower than PTC, no validated stiffness thresholds exist for MTC. Both SE and SWE demonstrate significant overlap between benign and malignant nodules, limiting diagnostic accuracy. The rarity of MTC further restricts the development of elastography-specific criteria, and interpretation is complicated by factors such as calcifications, lesion depth, and operator variability [12,14,15,16].

Diagnosing MTC can be difficult due to the overlap between its US features and those of benign nodules, alongside ongoing controversy surrounding the specificity of classic high-risk indicators. The limitations of conventional US, including subjective interpretation and operator variability, highlight the need for additional diagnostic tools. This study aims to explore whether elastographic stiffness measurements, when used in addition to conventional US features, can enhance diagnostic precision and improve the diagnostic accuracy of MTC.

## 2. Materials and Methods

### 2.1. Patient Inclusion and Exclusion Criteria

This retrospective observational study was conducted between January 2013 and June 2024 at a specialized endocrinology center in Timisoara, Romania (Dr. D Medical Center), spanning a total of 12 years. During this period, a total of 442 thyroid cancers were diagnosed, comprising 390 PTC, of which 121 were micro-PTC, 32 FTC, and 20 MTC. All patients were evaluated using conventional US and either SE or 2D-SWE.

The nodules evaluated during the specified period were selected for FNA based on classical US characteristics, following the American Thyroid Association (ATA) guidelines: low-risk nodules ≥ 2 cm, intermediate-risk nodules ≥ 1.5 cm, and high-risk nodules ≥ 1 cm. In high-risk nodules between 0.5 and 1 cm, FNA was performed only if individual or anamnestic risk factors were present [17]. Among the nodules assessed with conventional US, those that met these criteria and underwent FNA contributed to the thyroid cancer diagnoses recorded during the study period. Confirmed Bethesda II nodules with a visible goiter or objective compression, as well as nodules classified as Bethesda III–VI, were referred for surgery, and pathology reports were obtained.

This study included only cases of MTC confirmed by pathology, which was considered the gold standard for diagnosis. All included cases underwent both conventional US and elastography evaluation (SE or SWE) prior to surgery. When multiple nodules were described in the same patient, only the nodule that contributed to the final pathological diagnosis was analyzed.

Patients were excluded if they had any other type of thyroid cancer, lacked complete US or elastography evaluation, were examined in a different clinical setting, were not operated on, or did not provide a complete pathology report.

The study was approved by the Ethics Committee of Victor Babes University of Medicine and Pharmacy, Timisoara (approval no. 52/2 October 2023), and conducted in accordance with the ethical guidelines of the Helsinki Declaration. Written informed consent was obtained from all patients prior to their inclusion in the study.

### 2.2. Conventional US and Elastography Evaluation

Conventional B-mode thyroid US was conducted using two high-resolution US machines: the Hitachi Preirus (Hitachi Medical Corporation, Tokyo, Japan) equipped with a multifrequency linear probe (5–18 MHz) and the Aixplorer Mach 30 (SuperSonic Imagine, Aix-en-Provence, France) featuring an L18-5 linear probe (5–18 MHz) [18]. All examinations were performed by an experienced operator to ensure uniformity and reliability. Patients were positioned supine with their necks slightly hyperextended to optimize the anterior cervical exposure, supported by a pillow for stabilization. Generous amounts of coupling gel were applied, and minimal pressure was exerted to prevent compression artifacts.

Each thyroid nodule underwent a detailed evaluation, which included measurements of its transverse, longitudinal, and anteroposterior diameters, as well as volume. Additional features assessed included nodule shape (taller-than-wide or wider-than-tall), composition (solid or mixed), echogenicity (iso-, hyper-, hypo-, or markedly hypoechoic), homogeneity, margins (regular or irregular), calcifications (micro-, macro-, or absent), thyroid capsule integrity, and evidence of extracapsular invasion. Doppler ultrasound was used to describe the vascular patterns as predominantly perinodular, intranodular, or mixed; however, these characteristics were not incorporated into the malignancy risk assessment. The lateral cervical compartments were also evaluated for pathological lymph nodes [3,17,19]. All nodules were stratified into three risk categories based on conventional US and classified according to the European TIRADS: low risk (EU-TIRADS 2-3), intermediate risk (EU-TIRADS 4), and high risk (EU-TIRADS 5) [20].

Elastography was applied as a complementary technique, employing both SE and 2D-SWE [21]. SE was used to examine 12 out of the total 20 cases, with real-time imaging performed using the Hitachi Preirus device. During the examination, light, repetitive compression-decompression movements were applied to the transducer to generate a dynamic strain map. The system displayed a real-time feedback graph to ensure that the compression applied was within an optimal range (−0.5 to 0.5). A color-coded map was generated to differentiate tissue stiffness, with red indicating soft tissue, green for intermediate stiffness, and blue for hard tissue. Nodules were categorized based on the Asteria scoring system, which assigns an elasticity score (ES) from 1 to 4: 1—entirely soft lesion, 2—predominantly soft with a few stiff areas, 3—predominantly stiff with some soft regions, 4—completely stiff lesion. Nodules classified as ES 3 or 4 were considered high-risk. A semiquantitative strain ratio was also calculated, comparing the stiffness of the nodule with that of the surrounding thyroid parenchyma or adjacent tissue. The region of interest (ROI) was carefully selected to ensure consistency and avoid calcifications or artifacts [13,21].

2D-SWE was performed on the remaining 8 cases, using the Aixplorer Mach 30 machine, which employs advanced ultrafast imaging technology to generate supersonic shear waves. These waves allow for real-time imaging of tissue elasticity, displayed as a color-coded map where blue indicates soft tissue and red represents stiffer areas. To ensure reliable results, the probe was held steady, perpendicular to the skin, with only slight contact. Motion artifacts were minimized by instructing patients to remain still during image acquisition [22]. A box encompassing the nodule was adjusted for optimal elastogram visualization. Similarly to SE, a qualitative assessment was performed by categorizing the nodules into four color categories: 1—homogeneous, elastic (completely blue), 2—blue with vertical lines of green stiffness, 3—eccentric stiffness, 4—central stiffness with heterogeneity [23]. Nodules classified as ES 3 or 4 were considered high-risk. Subsequently, quantitative assessment was performed by placing a 2–4 mm sized ROI in the stiffest visible area of each nodule, avoiding calcifications and other artifacts. Both the maximum and mean elasticity indices (EIs), measured in kilopascals (kPa), were recorded after five repeated measurements in longitudinal views. Additionally, the QBox ratio, comparing the EI of the nodule to the surrounding parenchyma or adjacent muscle, was calculated. Increased stiffness documented on SWE (Emean > 30.5 kPa) was considered a high-risk feature for malignancy [21].

### 2.3. Cytological Examination and Pathology Report

Suspicious thyroid nodules were selected for FNA based on conventional US malignancy risk stratification, following the ATA guidelines [17]. Cytology results were reported according to the Bethesda system, with nodules categorized as Bethesda III–VI, as well as Bethesda II nodules associated with compressive symptoms or multinodular goiters with high-risk US features, being referred for surgery [3]. Histopathological examination of surgical specimens followed the World Health Organization (WHO) classification for thyroid tumors. To minimize bias, the cytologist interpreting FNA samples was blinded to the US findings, while the pathologists evaluating surgical specimens were blinded to both US and FNA results. For our study, we included only cases with histologically confirmed MTC.

### 2.4. Statistical Analysis

Data analysis was performed using MedCalc Statistical Software version 19.4 (MedCalc Software Ltd., Ostend, Belgium). Descriptive statistics were reported as mean, median, standard deviation, interquartile range (25–75%), minimum, and maximum for continuous variables and as percentages with raw counts for categorical variables. Group comparisons for non-normally distributed continuous variables (e.g., stiffness, size) were conducted using the Mann–Whitney U test. Correlations between continuous variables, such as serum calcitonin, nodule size, and elastographic parameters, were assessed using Spearman’s rank correlation coefficient. A *p*-value < 0.05 was considered statistically significant. Boxplots and scatter plots with regression lines were used to visually represent group differences and correlations.

## 3. Results

A total of 56 thyroid nodules were analyzed, of which 20 nodules corresponding to 20 patients with histologically confirmed MTC were included in the study. No cases of bilateral involvement were observed. Among these patients, the majority (70%) were female, with a median age of 53.5 years (range 18–80). The mean TSH level was 2.31 µIU/mL, with a relatively narrow interquartile range (1.33–2.87 µIU/mL), while FT4 levels varied more widely, with a mean of 11.35 pmol/L and values ranging from 0.83 to 26.04 pmol/L. Basal calcitonin levels showed substantial variability, with a mean of 218.53 pg/mL and a wide range (25–1223 pg/mL), reflecting the heterogeneous tumor burden in this cohort. Chronic autoimmune thyroiditis (CAT) was identified in 30% of cases. The baseline demographic and biochemical parameters of patients are presented in Table 1.

On US evaluation, the mean thyroid volume was 15.8 mL (range 6–35 mL), and patients had an average of 2.8 nodules per thyroid, with up to 7 nodules recorded. The largest nodule in each patient had a mean maximum diameter of 1.25 cm and a mean volume of 0.94 mL. Notably, 60% of the nodules were classified as suspicious according to TIRADS. Regarding high-risk US features, all nodules (100%) exhibited solid consistency and hypoechogenicity. Inhomogeneous echotexture was present in 80% of cases, while irregular margins were seen in 50%. Less frequent but notable features included taller-than-wide shape (15%), microcalcifications (15%), and macrocalcifications (20%). No nodules showed capsular disruption, and 30% of patients had suspicious cervical lymph nodes detected on US. Table 2 presents the ultrasonographic features of thyroid nodules identified in the MTC patients.

Elastographic evaluation revealed increased stiffness in MTC nodules. On qualitative SE, most nodules were rated as highly stiff (score 4). The mean strain ratio was 5.35, with values ranging from 1.35 to 7.8, indicating reduced compressibility compared to surrounding tissue. SWE also confirmed elevated stiffness, with a mean Emean of 42.4 kPa and a mean Emax of 59.3 kPa, reaching up to 118.5 kPa. The SWE ratio averaged 1.71, further supporting increased stiffness relative to normal parenchyma. Nodules were generally located at a mean depth of 2.05 cm. The elastographic characteristics of the thyroid nodules in MTC patients are summarized in Table 3.

**Figure 1:**
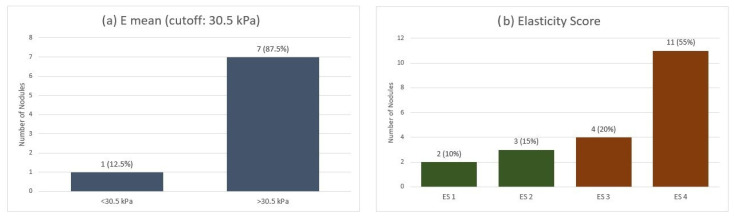
Elastographic evaluation of MTC nodules: (**a**) Quantitative stiffness assessment using shear-wave elastography (Emean cutoff: 30.5 kPa). (**b**) Qualitative strain elastography scores (ES 1–4). Emean—mean elasticity index; ES—elasticity score.

When applying the qualitative ES across the entire MTC group, we observed a mean ES of 3.2. Most nodules (55%) were classified as ES 4, indicating marked stiffness. In contrast, 20% were ES 3, 15% were ES 2, and only 10% were ES 1. Furthermore, on SWE quantitative analysis, using a cut-off value of 30.5 kPa for Emean, 7 out of 8 nodules exceeded this threshold (Figure 1).

**Figure 2:**
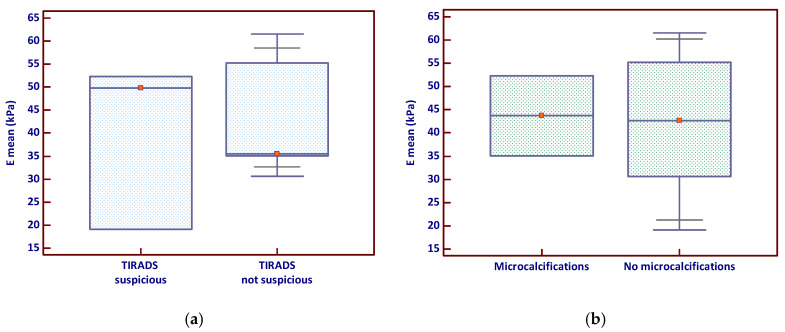
Emean stiffness in relation to (**a**) TIRADS suspicion and (**b**) microcalcifications (the red marker shows the median value). Emean—mean elasticity index; TIRADS—Thyroid Imaging Reporting and Data System.

The analysis of Emean by TIRADS classification showed that nodules classified as suspicious had a slightly higher median stiffness and a broader distribution of values (Figure 2a), suggesting greater variability in tissue stiffness among these lesions. In contrast, the comparison of Emean based on the presence of microcalcifications revealed no clear visual separation between groups, with overlapping interquartile ranges (Figure 2b); however, a few stiff nodules lacking microcalcifications appeared to elevate the overall mean.

**Figure 3:**
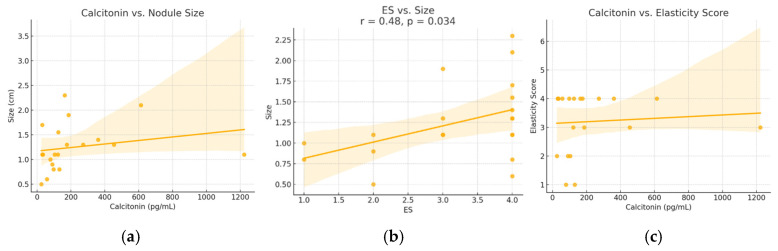
Relationships between (**a**) calcitonin versus nodule size, (**b**) elasticity score versus size and (**c**) calcitonin and elasticity score—in patients diagnosed with MTC. ES—elasticity score. Dots represent individual cases; the line shows the regression trend, and the shaded area indicates the 95% confidence interval.

A moderate and statistically significant positive correlation was identified between serum calcitonin levels and nodule size (Spearman r = 0.52, *p* = 0.018). A similar correlation was found between ES and nodule size (r = 0.48, *p* = 0.034). No significant correlation was observed between calcitonin levels and ES (r = 0.07, *p* = 0.768). These relationships are illustrated in Figure 3.

**Figure 4:**
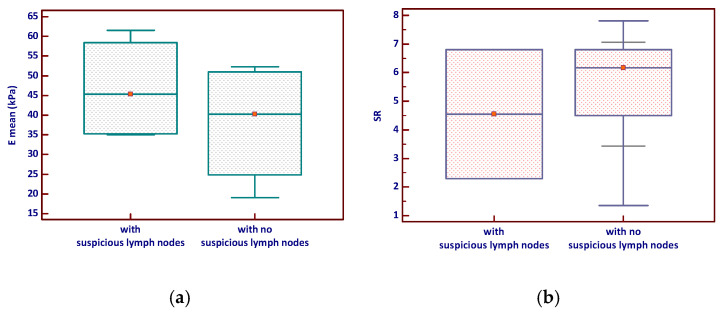
Elastography parameters—(**a**) E mean and (**b**) Strain ratio—according to lymph node status in MTC (the red marker shows the median value). Emean—mean elasticity index; SR—strain ratio.

Nodules associated with suspicious lymph nodes demonstrated higher mean SWE values (Emean) and a slightly broader range compared to those without suspicious nodes, although this difference was not statistically significant (*p* = 0.343). Similarly, the SR tended to be slightly higher in nodules with suspicious lymph nodes but with considerable overlap between groups and no significant difference (*p* = 0.964)—see Figure 4.

## 4. Discussion

MTC is a rare thyroid malignancy arising from parafollicular C cells, often characterized by early lymph node involvement and a variable US appearance. While US remains essential in the initial evaluation of thyroid nodules, the sonographic features of MTC frequently overlap with other malignancies and are not specifically addressed by RSSs such as TIRADS, which were primarily developed for differentiated thyroid cancers. High-risk features like solid composition, marked hypoechogenicity, irregular margins, and calcifications may be present, but their expression in MTC is inconsistent [7,8,9,10,11]. In this context, elastography has emerged as a promising adjunct tool, with preliminary evidence suggesting that MTC nodules often exhibit increased stiffness on both SE and SWE, potentially aiding in their identification and risk stratification [24,25].

In demographic terms, our findings align well with existing reports on MTC. At Pius Brinzeu Emergency University Hospital in Timisoara, over two separate two-year periods, pre-COVID and post-COVID, a total of 13 MTC cases were operated among 1505 total thyroidectomies and 456 confirmed thyroid cancers, yielding a rate of 2.8% MTC among thyroid cancers [26,27]. This incidence falls within the 1–5% range most epidemiological studies report [28]. Similarly, in our specialized endocrinology clinic, MTC comprised approximately 4.5% (20/442) of thyroid cancers, slightly on the higher end of the commonly cited range and likely reflecting our high-resolution US capabilities and referral bias toward more complex or suspicious cases. Finally, the demographic profile of our cohort, with a mean age in the early 50s and a female predominance, mirrors published data, where MTC typically presents around the fifth decade and shows a slight female preponderance [29].

In our study, MTC nodules consistently demonstrated several high-risk US features: all were solid and hypoechoic, 80% showed inhomogeneous echotexture, 50% had irregular margins, and calcifications (macro or micro) were present in approximately 35% of cases. Notably, only 15% exhibited a classic taller-than-wide orientation. These findings align closely with previous reports that observed solid composition in approx. 90%, hypoechogenicity in 84%, calcifications in 52%, and irregular margins in 64% of MTC cases [30,31]. Other multicenter studies also report up to one-third of MTC nodules lacking typical malignant sonographic features, resulting in as many as 26% being stratified as low-suspicion under current TIRADS/ATA systems [7,32]. In our cohort, 60% of nodules were classified as suspicious by TIRADS, mirroring the 50–65% classification rates previously described.

A key study evaluating vascularity, an element not included in our analysis, found that medullary nodules often display penetrating, branching blood flow patterns that distinguish low-suspicion MTC from benign lesions [33]. Our omission of Doppler vascular assessment represents a limitation, as this feature may enhance MTC detection in nodules that otherwise lack classic US characteristics. Nonetheless, our findings support the notion that while conventional US can reveal malignant traits in many MTC nodules, a significant proportion, perhaps 30–40%, could be misclassified or underappreciated if relying solely on TIRADS criteria, underscoring the need for multimodal evaluation including elastography and biochemical markers [34].

US elastography has been widely validated as a valuable adjunct to conventional B-mode imaging especially in PTC, demonstrating significant improvements in diagnostic accuracy. The WFUMB guideline reports sensitivity and specificity consistently above 80–90% for both SE and SWE in differentiating benign from malignant nodules [13,14]. One systematic review found combined qualitative SE sensitivity of 79% and specificity of 77%, while strain ratio techniques achieved 85% sensitivity and 80% specificity, with the area under the ROC curve (AUC) reaching 0.93 [12]. These robust metrics, commonly reported in PTC cohorts, have led many centers to adopt elastography, but its yield in MTC remains uncertain.

Contrary to the prior assumption that MTC may be an exception to this rule, our study indicates otherwise. MTC nodules in our cohort showed a mean qualitative ES of 3.2 (on a 1–4 scale), with 55% classified as ES 4, demonstrating significant stiffness. Quantitatively, 87.5% of nodules exceeded our institutionally validated SWE cutoff of 30.5 kPa (established in a PTC-focused study) [21]. These findings are consistent with prior studies reporting increased stiffness on SWE in MTC nodules, with mean elasticity values around 85.9 kPa, although still lower than PTC averages (>100 kPa) [24,25,35]. While SE may underperform in lesions with high amyloid content and low fibrosis, SWE offers quantitative rigor and reduced user dependence, making it a superior tool for detecting stiff MTC nodules in clinical practice. These findings suggest that SWE can serve as a valuable adjunct to conventional US, particularly when grayscale features are equivocal or do not meet TIRADS thresholds for FNA [24,25]. Given that MTC may present with less typical morphologic characteristics and thus fall below conventional biopsy criteria, incorporating stiffness values, such as the >30.5 kPa cutoff used in our study, can help refine diagnostic decision-making. In high-resolution centers equipped with advanced elastography, such thresholds may justify earlier FNA or calcitonin testing, even in nodules that do not meet standard size or TIRADS-based biopsy indications.

In our cohort, a moderate and statistically significant positive correlation was observed between serum calcitonin levels and nodule size (r = 0.52, *p* = 0.018), as well as between ES and nodule size (r = 0.48, *p* = 0.034), suggesting that larger MTC nodules tend to be both more secretory and stiffer. These results complement prior studies reporting that basal calcitonin levels reflect tumor burden and correlate with tumor size and staging in MTC [36,37]. However, we found no significant correlation between calcitonin levels and ES (r = 0.07, *p* = 0.768), indicating that tissue stiffness does not directly parallel secretory activity, an insight not extensively explored in earlier research, and it adds depth to our understanding of MTC pathophysiology.

Further supporting our findings, a recent study established a pre-operative basal calcitonin cutoff of 90 pg/mL for predicting lateral lymph node metastases [38]. In our study, 4 of the 6 patients with sonographically suspicious lymph nodes had calcitonin levels exceeding this cutoff, reinforcing the utility of calcitonin as a staging and risk marker that complements imaging findings and may support earlier intervention.

Moreover, we observed that nodules with sonographically suspicious lymph nodes tended to exhibit higher mean SWE values (Emean) and slightly elevated ES, although these differences did not reach statistical significance (*p* = 0.343 and *p* = 0.964, respectively). These trends mirror findings identifying an Emean > 37.5 kPa and E max > 66 kPa as independent predictors of lateral lymph node metastasis in MTC patients [39]. In our cohort, two nodules exceeded the Emean lymph node metastasis cutoff defined in that study, further suggesting elastographic stiffness may have clinical relevance in preoperative nodal assessment. However, only 8 of our 20 MTC cases underwent SWE evaluation, a limitation that underscores the need for larger, multicentric investigations. Despite the limited sample size, our data provide encouraging preliminary evidence that elevated stiffness in MTC nodules could correlate with nodal involvement and merit inclusion in combined imaging protocols for enhanced staging and management strategies.

Elastography, using both strain and shear-wave techniques, demonstrated compelling utility in identifying increased tissue stiffness within medullary nodules, challenging the long-held belief that MTCs are typically soft due to their amyloid-rich composition. In our study, 75% of nodules registered high stiffness (ES 3 or 4), and 87.5% exceeded the SWE Emean cutoff of 30.5 kPa. These results suggest elastography not only confirms stiffness in most MTC lesions but also performs comparably, or even slightly superior, to TIRADS in identifying high-risk cases. Additionally, elastography offers several clinical advantages: it is non-invasive, easily integrated into routine US protocols, and provides quantitative data (especially via SWE) to support decision-making. Its relative operator-independence (compared to SE or subjective TIRADS assessments) further increases its appeal as a robust adjunct in MTC suspicion.

Despite these strengths, our study has limitations that offer pathways for future research. First, only 40% of MTC nodules in our cohort underwent SWE evaluation, limiting the statistical power of our quantitative findings. The small sample size, inherent to the rarity of MTC, further restricts the robustness of statistical correlations and the generalizability of our results. The retrospective design and single-center nature of the study may also introduce selection bias, especially given our specialized endocrinology referral setting. We did not include vascularity evaluation, a known predictive feature for recurring/metastatic disease, which could enhance diagnostic models. Moreover, cutoffs like 30.5 kPa require external validation across multicentric cohorts for broader applicability. Future investigations should aim to standardize elastography protocols across high-resolution platforms, prospectively correlate stiffness data with histopathological markers (e.g., fibrosis, amyloid deposition), and incorporate simultaneous vascular and biochemical profiling to refine the predictive accuracy for nodal metastasis and clinical outcomes.

## 5. Conclusions

Our findings show that, contrary to the traditional perception of MTC as a soft tumor, the majority of nodules (75%) exhibited increased stiffness on elastography, particularly with high elasticity scores and Emean values exceeding the 30.5 kPa cutoff established for PTC. While elastography alone cannot replace cytology or biochemical testing, it offers valuable complementary data for risk stratification. Notably, elastography demonstrated slightly better performance than TIRADS in identifying high-risk nodules, despite all cases presenting with similar gray-scale features such as solid and hypoechoic structure. These results suggest that integrating elastographic assessment with TIRADS-based evaluation may enhance diagnostic confidence in suspected MTC cases. However, serum calcitonin levels and FNA remain indispensable for definitive diagnosis.

## Figures and Tables

**Table 1:** Basic patient characteristics of the MTC group.

	Mean/Percentage	Median	Std Dev	25–75%	Min	Max
Gender (female)	70%	-	-	-	-	-
Age	51.7	53.5	16.68	40.0–60.0	18	80
TSH	2.31	2.46	1.05	1.33–2.87	0.62	4.1
FT4	11.35	14.06	7.41	1.45–15.56	0.83	26.04
Calcitonin	218.53	123.5	281.65	72.9–207.5	25	1223
CAT	30%	-	-	-	-	-

CAT—chronic autoimmune thyroiditis; Min—minimum value; Max—maximum value.

**Table 2:** US characteristics of the thyroid nodules in MTC patients.

		Mean/%	Median	Std Dev	25–75%	Min	Max
Thyroid volume		15.8	15	7.783924	11.0–19.25	6	35
Number of nodules		2.8	3	1.542384	1.75–4.0	1	7
Max Dimension (nodule)		1.2475	1.1	0.473363	0.975–1.4375	0.5	2.3
Volume (nodule)		0.9375	0.55	0.950462	0.275–1.175	0.1	3.17
TIRADS (suspicious)		60% (12/20)	-	-	-	-	-
High-risk ultrasound characteristics	Solid consistency	100% (20/20)	-	-	-	-	-
	Hypoechogenicity	100% (20/20)	-	-	-	-	-
	Inhomogeneity	80% (16/20)	-	-	-	-	-
	Taller-than-wide shape	15% (3/20)	-	-	-	-	-
	Irregular margins	50% (10/20)	-	-	-	-	-
	Interrupted capsule	0% (0/20)	-	-	-	-	-
	Secondary lymph nodes	30% (6/20)	-	-	-	-	-
	Macrocalcifications	20% (4/20)	-	-	-	-	-
	Microcalcifications	15% (3/20)	-	-	-	-	-

TIRADS—Thyroid Imaging Reporting and Data System; Min—minimum value; Max—maximum value.

**Table 3:** Elastography characteristics of the thyroid nodules in MTC patients.

		Mean	Median	Std Dev	25–75%	Min	Max
Strain elastography	Qualitative score	3.2	4	1.056309	2.75–4.0	1	4
	Strain ratio	5.35	6.175	2.174229	3.9875–6.8	1.35	7.8
Shear-wave elastography	E mean	42.385	42.65	14.46543	33.96–53.025	19.1	61.5
	E max	59.28375	45.65	32.69635	37.9525–80.5	23.8	118.5
	SWE ratio	1.7125	1.65	0.581715	1.2–1.975	1.1	2.8
	Depth	2.05	1.85	0.939605	1.45–2.6	0.8	3.5

SWE—shear-wave elastography; Min—minimum value; Max—maximum value.

## Data Availability

The raw data supporting the conclusions of this article will be made available by the authors on request.
